# Longitudinal Profiling of the Tissue-Specific Expression of Genes Related with Insulin Sensitivity in Dairy Cows during Lactation Focusing on Different Fat Depots

**DOI:** 10.1371/journal.pone.0086211

**Published:** 2014-01-21

**Authors:** Behnam Saremi, Sarah Winand, Paula Friedrichs, Asako Kinoshita, Jürgen Rehage, Sven Dänicke, Susanne Häussler, Gerhard Breves, Manfred Mielenz, Helga Sauerwein

**Affiliations:** 1 Institute of Animal Science, Physiology and Hygiene Unit, University of Bonn, Bonn, Germany; 2 Clinic for Cattle, University of Veterinary Medicine Hannover, Foundation, Hannover, Germany; 3 Institute of Animal Nutrition, Friedrich-Loeffler-Institute (FLI), Federal Research Institute for Animal Health, Braunschweig, Germany; 4 Department of Physiology, University of Veterinary Medicine Hannover, Foundation, Hannover, Germany; Wageningen University, Netherlands

## Abstract

In dairy cows the milk associated energy output in early lactation exceeds the input via voluntary feed intake. To spare glucose for mammary lactose synthesis, peripheral insulin sensitivity (IS) is reduced and fat mobilization is stimulated. For these processes a link between IS and the endocrine functions of adipose tissue (AT) is likely; we thus aimed to characterise the mRNA expression from bovine AT derived proteins and receptors that are related to IS according to the literature in metabolically active tissues plus systemic IS throughout lactation. Conjugated linoleic acids (CLA) reduce milk fat thus decreasing the milk drain of energy and potentially dampening lipolysis, but may also affect IS. Subcutaneous (s.c.) AT and liver from pluriparous cows receiving either control fat or CLA supplement (100 g/day from 1 to 182 days in milk each) were biopsied covering week −3 to 36 relative to parturition. In an additional trial with primiparous cows treated analogously and slaughtered on days in milk 1, 42 or 105, samples from liver, udder, skeletal muscle and 3 visceral and 3 s.c. AT were obtained and assayed for mRNA abundance of adiponectin, its receptors, leptin, leptin receptor, PPARγ, PPARγ2, IL-6, and TNF-α. In pluriparous animals, the mRNA abundance of most of the target genes decreased after parturition in s.c. AT but increased in liver. In primiparous cows, AT depot specific differences were mostly related to retroperitoneal AT; adiponectin receptor 1 and TNF-α were affected predominantly. CLA effects in primiparous cows were largely limited to decreased PPARγ2 mRNA abundance in udder tissue. In pluriparous cows, insulin secretion was increased by CLA resulting in decreased systemic IS but without consistent changes in tissue target mRNA abundance. The temporal gene expression profiles from the adipokines and related receptors support their coactive function in adapting to the needs of lactation.

## Introduction

Late pregnancy and lactogenesis are linked to comprehensive endocrine and metabolic changes. Homeorhetic adaptations are necessary to accomplish the nutrient drain towards the mammary gland. They are associated with decreasing peripheral insulin sensitivity (IS) in peripheral tissues other than the mammary gland in dairy cattle [1] just as in other mammalian species like humans [2]. Nevertheless, genetic selection for milk synthesis in dairy cattle resulted in a particular distinctive and prolonged negative energy balance (NEB) which is often associated with metabolic diseases like fatty liver [3].

Messenger molecules derived from adipose tissue (AT), i.e. adipokines, are important metabolic regulators and modulate IS [4]. This is well known for monogastric species in contrast to ruminants. The adipokines are related to energy metabolism (e.g. adiponectin (ADIPOQ) and leptin (LEP) and to inflammation (e.g. tumor-necrosis factor-α (TNF-α) and interleukin-6 (IL-6)) [5]. LEP and ADIPOQ increase IS whereby LEP is positively and ADIPOQ is negatively linked with body fat content [6], [7]. LEP mainly exerts its effects through the leptin receptor (LEPR) isoform b (LEPRB) [8] and ADIPOQ via ADIPOQ receptor 1 (ADIPOR1) and ADIPOQ receptor 2 (ADIPOR2), as observed in humans [9]. Adiponectin reduces gluconeogenesis and decreases hepatic glucose release. Beta-oxidation of fatty acids is increased by ADIPOQ in liver and skeletal muscle. In the latter glucose uptake is also improved [7], [9]. LEP is negatively associated with energy intake. It stimulates beta-oxidation and reduces blood glucose and blood lipids [6]. Activation of inflammatory pathways in AT and release of inflammatory cytokines from AT can affect IS directly at the level of insulin sensing, e.g. reduced phosphorylation of insulin receptor and insulin receptor substrate-1 lead to decreased glucose uptake as shown for TNF-α [10] and IL-6 [11].

The transcription factor peroxisome proliferator-activated receptor gamma (PPARγ) is a central regulator of genes related to insulin sensitivity, including ADIPOQ, IL-6 and TNF-α [12], [13]. In dairy cattle it was shown that PPARγ gene expression is linked to lipogenic gene expression in the mammary gland [14] and that the PPARγ agonist 2,4-thiazolidinedione increases plasma glucose [15]. In cattle, two isoforms of PPARγ (PPARγ1 and PPARγ2) sharing 90% identity are described [16]. In general, PPARγ2 is the predominant isoform in AT; PPARγ plays an important role in adipocyte differentiation and improves IS e.g. by increasing glucose uptake and by the induction of genes related to fatty acid metabolism and fatty acid uptake. In addition, the isoform PPARγ2 is needed for adiponectin gene transcription [17].

Fat from different areas of the body displays distinct structural and functional properties including adipokine secretion. In humans and rodents, visceral (v.c.) AT feature higher lipogenic and lipolytic activities and produce more pro-inflammatory cytokines, while subcutaneous (s.c.) AT are the main source of LEP and ADIPOQ, and thus have disparate roles, amongst others in IS as reviewed in [18]. So far, studies in dairy cows were mostly limited to s.c. fat which can readily be biopsied.

Linoleic acid and their derivatives like conjugated linoleic acids (CLA) are ligands for PPARs [19], [20]. In 3T3L1 adipocytes *trans-*10, *cis-*12 CLA decreases ADIPOQ in both PPARγ dependent and independent mechanisms *in vitro* [21]. CLA is also a ligand for free fatty acid receptor 1 and activation of this receptor increases glucose stimulated insulin secretion in mice [22]. From rodent studies using high doses (up to 1% of the diet) of *trans-*10, *cis-*12 CLA, dramatic loss of AT, inflammation, insulin resistance and hepatic steatosis are reported [23]; however, when using lower doses, the effects were largely limited to reduced AT lipogenesis. In dairy cattle, CLA may act energy-sparing as the *trans-*10, *cis-*12 CLA isomer is related to milk fat depression whereas whole body fat seems largely unaffected during both short and long-term treatments with this CLA isomer [24]. Milk fat depression may improve NEB in dairy cows but the effects are not conclusive yet as discussed by Hötger et al. [25]. This group has shown that the main isomers (*trans-*10, *cis-*12 CLA, *cis-*9, *trans-*11 CLA) decrease endogenous glucose production and milk fat synthesis but increase plasma glucose, whereby insulin secretion in cows during their second lactation was not affected.

The understanding of the expression and regulation of IS related genes in relevant tissues involved in energy metabolism in dairy cattle may help to develop strategies to reduce the incidence for metabolic diseases in future. Therefore we aimed to characterise the time course of the expression of IS related genes during lactation in different AT of v.c. and s.c. localisation in addition to muscle, liver and mammary gland. Based on the potential of CLA to spare energy in addition to affect glucose metabolism and possibly IS, we supplemented primiparous and pluriparous Holstein cows' diet with a 1:1 mixture of *trans-*10, *cis-*12 CLA and *cis-*9, *trans-*11 CLA which is registered as feed supplement in the EU. The main focus aims were (1) to characterise the temporal expression pattern in s.c. AT and liver biopsies from pluriparous cows from late pregnancy to late lactation, (2) to compare the mRNA expression between different s.c. and v.c. AT depots and in other metabolically relevant tissues available through timed slaughter of primiparous cows and (3) to test for potential CLA effects in cows of both parity groups. The target genes we selected herein are related to IS, as substantiated by a vast body of literature, and we therefore aimed to test not only tissue mRNA expression but also to estimate systemic IS using the “Revised Quantitative Insulin Sensitivity Check Index” (RQUICKI) that was suggested for use in dairy cows [26]. We aimed to identify the AT depots that are mostly affected throughout lactation and to define the predominant genes out of the analysed panel of genes involved in the regulation IS throughout lactation.

## Materials and Methods

All animals were housed in free stall barns at the experimental station of the Friedrich Loeffler Institute, Federal Research Institute for Animal Health, Braunschweig, Germany.

### Ethics statement

The experiments were approved by the competent authority, the lower saxony state office for consumer protection and food safety (LAVES, file no. 33.11.42502-04-071/07, Oldenburg, Germany). The regulations of the German Animal Welfare Act (TierSchG) in its respective edition were met.

### Animals, diets and treatments

Trial 1. German Holstein pluriparous cows (n = 21) were fed a partial mixed ration consisting of 38% grass silage, 25% corn silage and 37% concentrate on a dry matter basis for ad libitum intake over the entire experimental period. Diets were formulated according to the recommendations of the German Society of Nutrition Physiology [27]. More details about the composition of the diet, the experiment, and the outcomes in terms of performance (including the calculated energy balance), metabolite concentrations and milk FFA profile from this experiment are described by Pappritz et al. [28]. On day 1 in milk (DIM) 1 the animals were allocated to 2 groups: a CLA group (n = 11) and a control group (n = 10). During the supplementation period (from 1 to 182 DIM), the animals received 4 kg of additional concentrate containing the fat supplements. The animals of the CLA group received 100 g/d encapsulated CLA (Lutrell Pure, BASF, Ludwigshafen, Germany). The control group received 100 g/d of a control fat supplement (Silafat, BASF) in which CLA were substituted by stearic acid to form an isoenergetic control diet using a fatty acid with the same number of carbon atoms as in CLA. In the CLA group, the animals consumed 7.6 g/day each of the *trans*-10, *cis*-12 and *cis*-9, *trans*-11 CLA isomers. Liver and s.c. AT from tail head were biopsied on d 21 before calving and on DIM 1, 21, 70, 105 182 196, 224, and 252. The biopsy procedures were previously described in detail by Saremi et al. [29]. For gene expression analyses, samples were restricted to the pluriparous cows of the control group (all sampling dates) and of the CLA group (d −21, 21, 105, 196, and 252) as the focus of the study was related to changes throughout lactation and the capacity of handling the samples was limited. Jugular blood samples were collected on d –21, –14, –7, 1, 7, 14, 21, 35, 49, 70, 105, 140, 182, 189, 196, 210, 224, 238, and 252 relative to calving.

Trial 2. German Holstein primiparous cows (n = 25, average age at calving was 23 months) were fed a partial mixed ration (ad libitum) according to the recommendations of the German Society of Nutrition Physiology [27]. The ration consisted of 63% silage (60% corn silage, 40% grass silage) and 37% concentrate on a dry matter basis. Five cows were slaughtered on 1 DIM and the remaining animals (n = 20) were randomly allocated to either receive 100 g/d of the control fat supplement (Silafat, BASF) or of CLA (Lutrell Pure, BASF) starting from 1 DIM. All experimental details and main outcomes in terms of performance, adipose depot and organ weights, body composition, body fat mobilization, protein accretion, and energy utilization were reported by von Soosten et al. [30], [31]. Five cows per group were slaughtered at 42 and 105 DIM. Samples from 3 v.c. AT (omental, mesenteric, and retroperitoneal), 3 s.c. AT (tail head, withers, and sternum), liver, *M. semitendinosus*, pancreas, and mammary gland parenchyma were taken about 25 min after stunning. Blood samples of about 40 mL were collected from *V. jugularis* after the morning milking on d –21, –14, –7, –3, 1 (n = 25), 7, 14, 28, 42 (n = 20), and 105 (n = 10) relative to parturition.

The tissue samples from both trials were snap-frozen in liquid nitrogen and stored at –80°C for RNA extraction and analysis. The blood samples were collected in different tubes to obtain sodium fluoride-EDTA plasma, heparin plasma or serum. Plasma samples were stored at –20°C and sera at –80°C until analysed.

### Relative quantification by real-time PCR

Preparation of samples including RNA extraction and cDNA synthesis together with real-time PCR and reference genes characteristics, selection, and measurements are described in detail by Saremi et al. [29], [32]. Relative quantification of the target genes was performed as summarized in Table 1. Amplicon standard curve was used except for PPARγ*2* in Trial 1 and 2 and LEPRB and TNF*-α* in Trial 1 where cDNA standards curve was used. The standard curve was used to correct data based on PCR efficiency per run. Two µL cDNA (diluted 1 to 4) as template and 5 µL SYBR Green JumpStart Taq Readymix (Sigma-Aldrich, Steinheim, Germany) in a total volume of 10 µL were run in an Mx3000P real-time PCR cycler (Stratagene, Amsterdam, Netherlands and Agilent, Santa Clara, CA). Based on biological (abundance) and/or technical reasons (total RNA in use for cDNA synthesis), not all genes were quantified in each tissue in Trial 1 (ADIPOQ and PPARγ2 in liver, IL-6 and LEPRB in s.c. AT were not evaluated) and in Trial 2 (ADIPOQ in liver and muscle, PPARγ in muscle and mammary gland, LEP in liver were not evaluated).

**Table 1:** Characteristics of the primers and the real-time PCR conditions.

Primers	Sequence (5′-3′)	NIH Genbank accession number	bp^11^	Concen- tration (nM)	Mean Cq^12^ value^13^	Annealing^14^ (sec/°C)
*ADIPOQ* 1						
Forward	CTGGAGAGAAGGGAGAGAAAG	NM_174742	204	800	24.9	75/60
Reverse	TGGGTACATTGGGAACAGTG					
*ADIPOR1* 2						
Forward	GCTGAAGTGAGAGGAAGAGTC	XM_593692	118	800	22.3	45/60
Reverse	GAGGGAATGGAGTTTATTGCC					
*ADIPOR2* 3						
Forward	GGCAACATCTGGACACATC	XM_580459	200	400	21.5	45/60
Reverse	CTGGAGACCCCTTCTGAG					
*LEP* 4						
Forward	GACATCTCACACACGCAG	U62123	183	400	25.4	30/60
Reverse	GAGGTTCTCCAGGTCATT					
*LEPRB* 5						
Forward	ACCACACCTTCCGTTCTCAG	AB199589	164	400	28.8	30/60
Reverse	GGGACAACACTCTTGACTC					
*LEPR* 6						
Forward	CCACTGTTGCTTTTGGAGCGAGGA	NM_001012285	125	100	23.0^15^	60/62
Reverse	TGTTCCAGTTTGCACCTGTTTGCT					
*PPARγ2* 7						
Forward	ATTGGTGCGTTCCCAAGTTT	Y12420	57	400	21.0	60/60
Reverse	GGCCAGTTCCGTTCAAAGAA					
*PPARγ* 8						
Forward	AGGATGGGGTCCTCATATCC	Y12420	121	800	25.3	60/61
Reverse	GCGTTGAACTTCACAGCAAA					
*IL-6* 9						
Forward	TGCAGTCTTCAAACGAGTGG	BC123577	182	400	26.6	60/30
Reverse	TAAGTTGTGTGCCCAGTGGA					
*TNF-α* 10						
Forward	TGCCTGCTGCACTTCGGGGTA	EU276079	50	800	28.8	60/60
Reverse	CCTGGGGACTCTTCCCTCTGGGG					

^1^ Adiponectin, [45].

^2^ Adiponectin receptor 1, [45].

^3^ Adiponectin receptor 2, [45].

^4^ Leptin, [93].

^5^ Leptin receptor isoform b, [45].

^6^ Leptin receptor.

^7^ Peroxisome proliferator-activated receptor *gamma2.*

^8^ Peroxisome proliferator-activated receptor *gamma*, [94].

^9^ Interleukin-6.

^10^ Tumor necrosis factor-α.

^11^ bp: base pairs.

^12^ Cq: Quantification cycle.

^13^ Based on slaughter experiment.

^14^ Initial denaturation  = 10 min at 95°C; denaturation  = 30 s at 95°C; extension  = 30 s at 72°C, except for *TNF-α*, *PPAR*γ2, *PPAR*γ, *LEPR*, and *LEPRB* (60 s at 72°C).

^15^ Based on liver and s.c. fat biopsies from Trial 1.

### Reference gene stability and data analysis

In each individual case, comparing treatment effects within a single tissue or comparing different tissues, an adequate panel of reference genes was selected according to Saremi et al. [29], [32].

Trial 1. The reference genes used were low density lipoprotein receptor-related protein 10 (LRP10), glyceraldehyde-phosphate-dehydrogenase (GAPDH), and RNA Polymerase II (POLR2A) for s.c. AT and LRP10, POLR2A, and eukariotic translation initiation factor 3, subunit K (EIF3K) for liver tissue according to Saremi et al. [29]. The geometric mean of the reference gene abundances was used for normalisation. Data are presented as ratio of the copy numbers of genes of interest and the geometric mean of the corresponding reference genes.

Trial 2. The quantification cycle values were imported in qBASE^plus^ version 2.0 (Biogazelle, Ghent, Belgium) and all subsequent calculations and data quality controls were done based on this software [33]. Reference genes were tested per tissue and the lowest V value obtained for the number of reference genes needed for normalisation as described by Saremi et al. [32]. Briefly, for normalisation of the efficiency corrected data the reference genes EIF3K, LRP10, POLR2A, Emerin, Marvel domain containing 1 and Hippocalcin-like 1 were selected and used, depending on the tissues being compared [32] were used. The data used for statistics was generated by geNorm^plus^ as a part of qBASE^plus^ version 2.0 (Biogazelle, Ghent, Belgium).

### Analysis of non esterified fatty acids, ß-hydroxy butyric acid, LEP, and insulin in blood samples

Glucose was measured in sodium fluoride-EDTA plasma, for quantification of insulin, non esterified fatty acids (NEFA), and ß-hydroxy butyric acid (BHBA) heparinised plasma was used. The plasma concentrations of glucose, NEFA, and BHBA were determined by an automatic analyser (Cobas Mira Plus System from Roche Diagnostica Ltd, Basel, Switzerland) using commercial test kits (Glucose: Glucose Hexokinase Fluid 5+1, MTI Diagnostics GmbH, Idstein, Germany; NEFA: HR(2) R1+R2 Set, WAKO Chemicals GmbH, Neuss, Germany; BHBA: RANBUT, RB 1008, Randox Laboratories GmbH, Wülfrath, Germany). LEP concentrations were determined in serum by ELISA [34]. The intra and inter assay coefficients of variations were 6.3% and 13.9%, respectively, the limit of detection was 0.3 ng/mL. The concentrations of insulin in plasma were measured using a commercially available double antibody radioimmunoassay (DSL-1600, Diagnostic Systems Laboratories, Inc., TX). The intra and inter assay coefficients of variation were 6.3% and 8.8%, respectively.

### Calculations and statistical analyses

The RQUICKI was calculated based on the concentrations of plasma glucose, NEFA, and insulin [35] as previously used to estimate IS in dairy cows [26] by the following equation:

All statistical analyses were performed using SPSS (version 20, SPSS Inc., Chicago, IL). For Trial 1, the mixed model procedure was used. Treatment (control and CLA) was considered as fixed factor, sampling dates (time) as repeated effect, and the respective interaction was included in the model. For serum LEP, but not for mRNA analyses which were limited to the cows from Trial 1, parity (cows or heifers) was considered as fixed effect together with its interaction with treatment. For each time series the prepartum value was considered as a covariate in the model to check for the effect of possible differences between treatment groups prepartum. The covariate was never significant. The covariance structures compound symmetry, unstructured and heterogeneous first-order autoregressive were tested; latter was used for serum data while compound symmetry was used for mRNA data. Bonferroni correction was used for correcting multiple comparisons.

For Trial 2 in which the repeated design for tissue samples was not applicable, all data were tested for homogeneity of variances (*P*≤0.1). Accordingly the general linear model with the fixed effects of treatment, dates, and the respective interaction, or non parametric tests (Kruskal-Wallis or Mann-Whitney) were applied (*P*≤0.05). For correlation analyses Pearsons product-moment coefficients were calculated, presentation of the coefficients of correlations is limited to significant correlations *(P*≤0.05). All data are presented as mean ± SEM. For both trials *P*≤0.05 was considered significant.

## Results

### Trial 1

**Figure 1:**
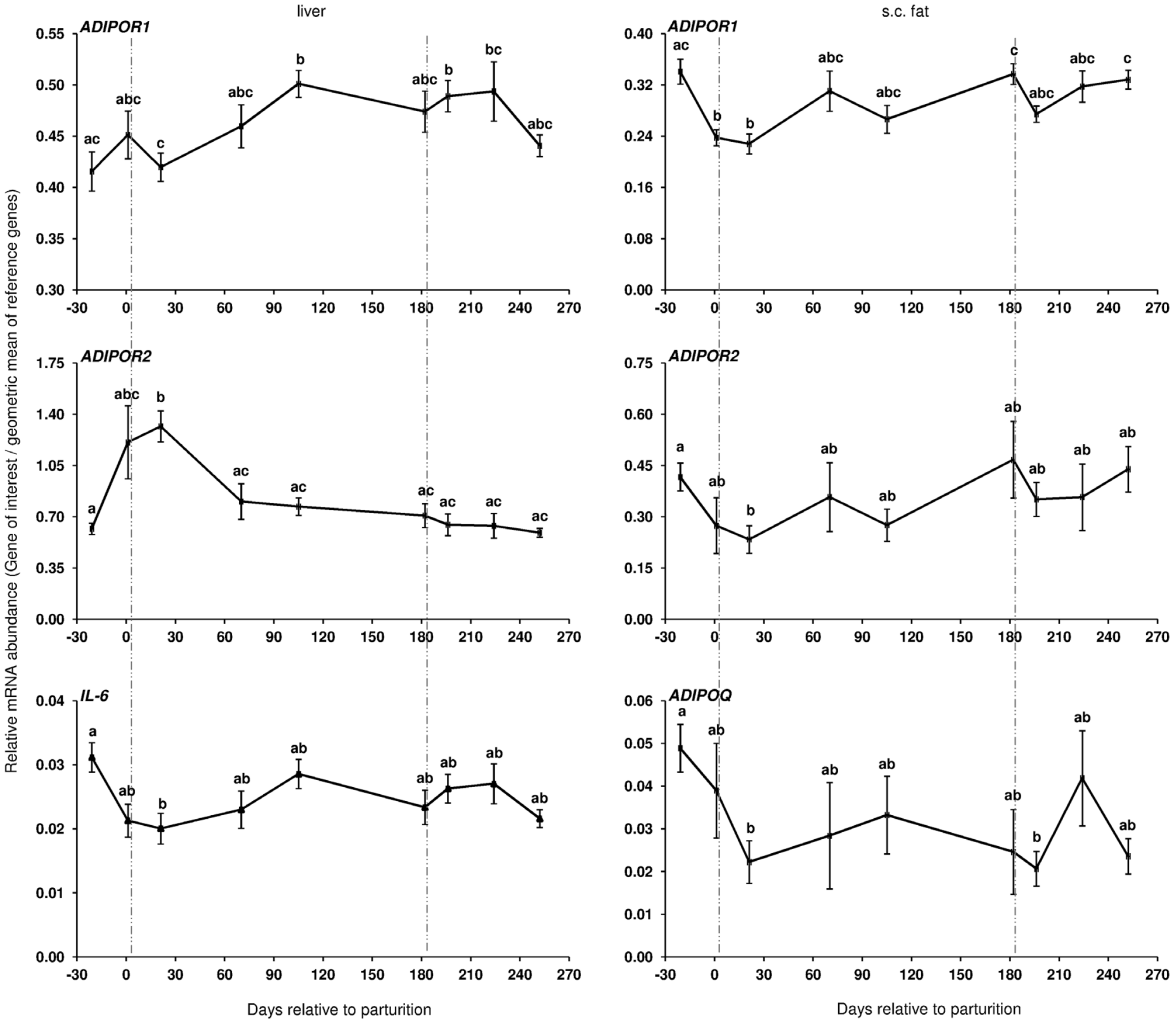
Longitudinal mRNA expression of genes related to insulin sensitivity in pluriparous cows during lactation I. Longitudinal mRNA expression of adiponectin receptors (ADIPOR1 and ADIPOR2) in liver and s.c. tail head biopsies of dairy cows together with adiponectin (ADIPOQ) and interleukin-6 (IL-6) mRNA abundance in s.c. adipose tissue and in liver of Trial 1, respectively. Cows were fed with conjugated linoleic acids (CLA, Lutrell® Pure, BASF SE, Ludwigshafen, Germany) at 100 g/day CLA or a control fat supplement (Silafat®, BASF SE) from day 1 until day 182 postpartum in Trial 1. Primiparous cow samples are not included (There are no sample in the CLA group at days 1, 70, 182, and 224) [Control: n = 10, CLA: n = 11]. Cumulative mRNA expression of both control and CLA is shown because CLA effect was insignificant. For normalisation, lipoprotein receptor-related protein 10 (LRP10), RNA Polymerase II (POLR2A) and eukariotic translation initiation factor 3 (EIF3K) in liver and LRP10, glyceraldehyde-phosphate-dehydrogenase (GAPDH), and POLR2A in s.c. adipose tissue were used as reference genes. Different letters indicate significant differences between days relative to parturition (*P*≤0.05; mean ± SEM). Area between vertical lines corresponds to the CLA supplementation period.

**Figure 2:**
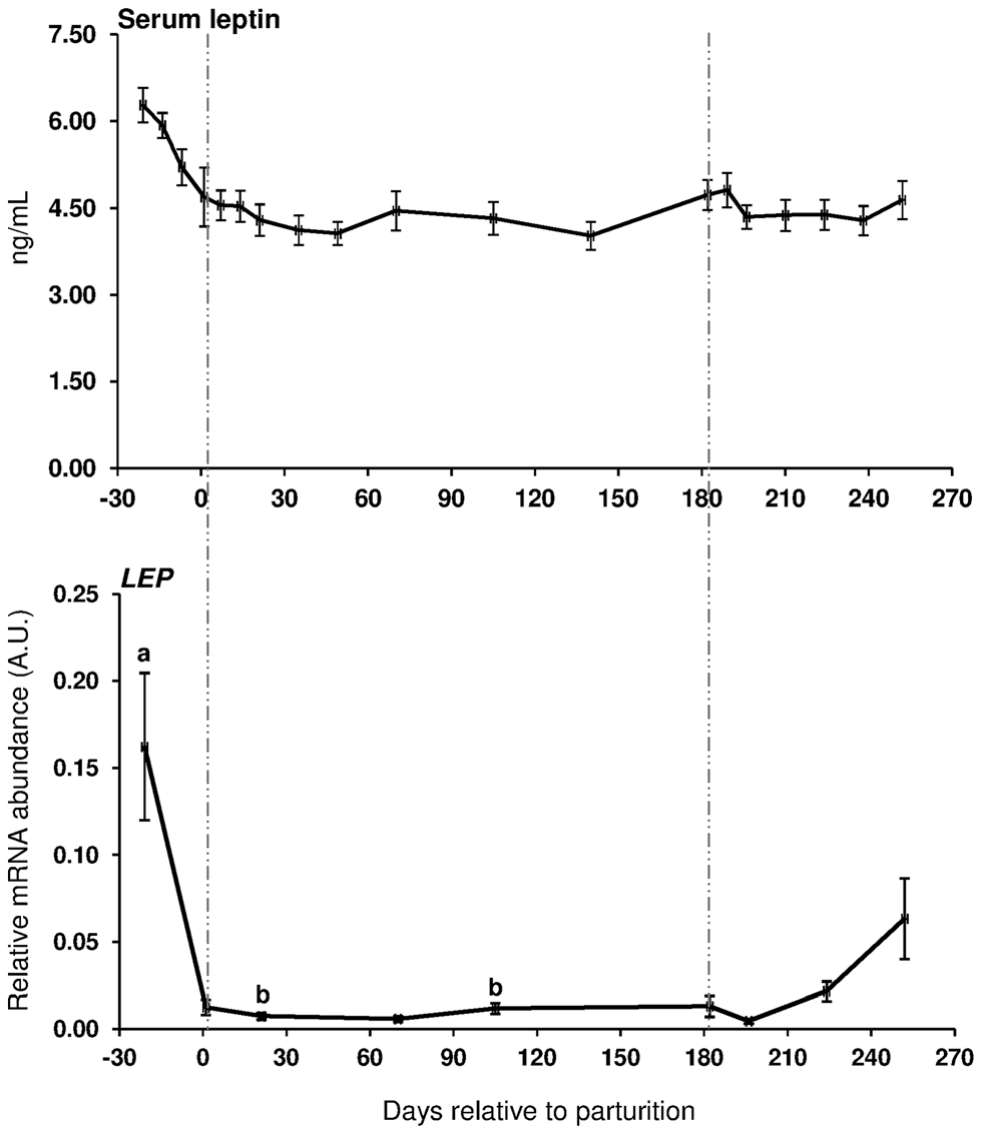
Serum leptin in primiparous and pluriparous as well as leptin mRNA data (LEP) in pluriparous cows. Leptin serum concentrations (upper graph) and leptin mRNA abundance in s.c. tail head adipose tissue (lower graph) in pluriparous and primiparous cows receiving conjugated linoleic acids (CLA, Lutrell® Pure, BASF SE, Ludwigshafen, Germany) at 100 g/day (CLA) or a control fat supplement (Silafat®, BASF SE) from day 1 until day 182 postpartum in Trial 1. All animals and samples were included for serum data [Control: pluriparous cows n = 10, primiparous cows n = 5; CLA: pluriparous cows n = 11, primiparous cows n = 5]. For mRNA data only pluriparous cow samples from days −21, 1, 21, 70, 105 182 196, 224, and 252 relative to parturition for control group and days −21, 21, 105, 196, and 252 for CLA group were analysed. For normalisation, lipoprotein receptor-related protein 10 (LRP10), RNA Polymerase II (POLR2A) and glyceraldehyde-phosphate-dehydrogenase (GAPDH) in s.c. adipose tissue were used as reference genes. ns: not significant. Different letters indicate significant differences between days relative to parturition (*P*≤0.05; mean ± SEM). Area between vertical lines corresponds to the CLA supplementation period. A.U.: arbitrary units.

**Figure 3:**
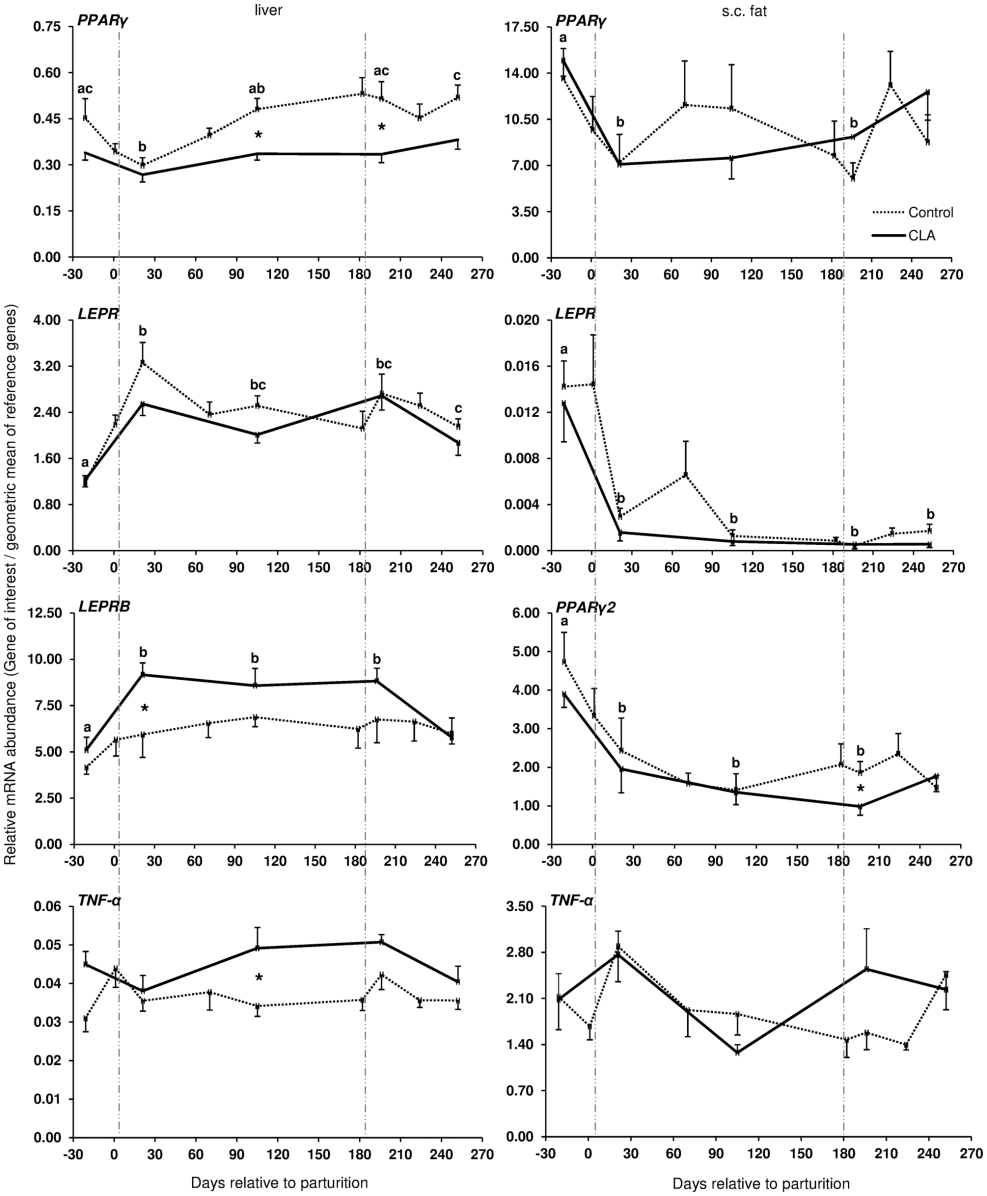
Longitudinal mRNA expression of genes related to insulin sensitivity in pluriparous cows during lactation. Longitudinal mRNA expression of peroxisome proliferator-activated receptor (PPAR) *γ* and *γ2*, leptin receptor (LEPR), leptin receptor isoform b (LEPRB), and tumor necrosis factor (TNF)*-α* in liver and s.c. tail head adipose tissue biopsies of dairy pluriparous cows fed with conjugated linoleic acids (CLA, Lutrell® Pure, BASF SE, Ludwigshafen, Germany) at 100 g/day CLA or a control fat supplement (Silafat®, BASF SE) from day 1 until day 182 postpartum in Trial 1(defined by vertical lines). Primiparous cow samples are not included. There are no sample in the CLA group at days 1, 70, 182, and 224 [Control: n = 10, CLA: n = 11]. For normalisation, lipoprotein receptor-related protein 10 (LRP10), RNA Polymerase II (POLR2A) and eukariotic translation initiation factor 3 (EIF3K) in liver and LRP10, glyceraldehyde-phosphate-dehydrogenase (GAPDH), and POLR2A in s.c. adipose tissue were used as reference genes. Different letters indicate significant differences between days relative to parturition where there are differences otherwise no letter used. CLA effects are defined using asterisks (*P*≤0.05; mean ± SEM). Area between vertical lines corresponds to the CLA supplementation period.

During the transition from late pregnancy to early lactation (transition period) in pluriparous cows, the mRNA abundance of the target genes was decreased in the majority of the cases in s.c. AT (Figures 1, 2, and 3). In detail, the mRNA abundance for LEP, LEPR, ADIPOQ, ADIPOR1, ADIPOR2, PPARγ, and PPARγ2 was decreased at that time in s.c. AT. The mRNAs for PPARγ, PPARγ2 and LEPR in s.c. AT remained lower throughout the analysed time period up to 252 DIM as compared to ante partum (a.p.). Similarly, the amount of PPARγ and IL-6 mRNA in liver was reduced. In contrast, the quantity of LEPR and LEPRB as well as ADIPOR2 mRNA in liver increased during the transition period. The abundance of LEPR and LEPRB mRNA in liver was higher up to day 196 DIM than a.p., and decreased thereafter to values comparable to the situation a.p., but remained higher at day 252 in case of LEPR. The mRNA quantity for ADIPOR2 in liver was higher at 21 DIM vs. −21 DIM and decreased to the end of the analysed period. No peripartal and lactation related differences were observed for TNF-α in liver and s.c. AT. The pattern of serum LEP followed roughly the mRNA abundance throughout the transition period but these changes were not significant (Figure 2).

**Figure 4:**
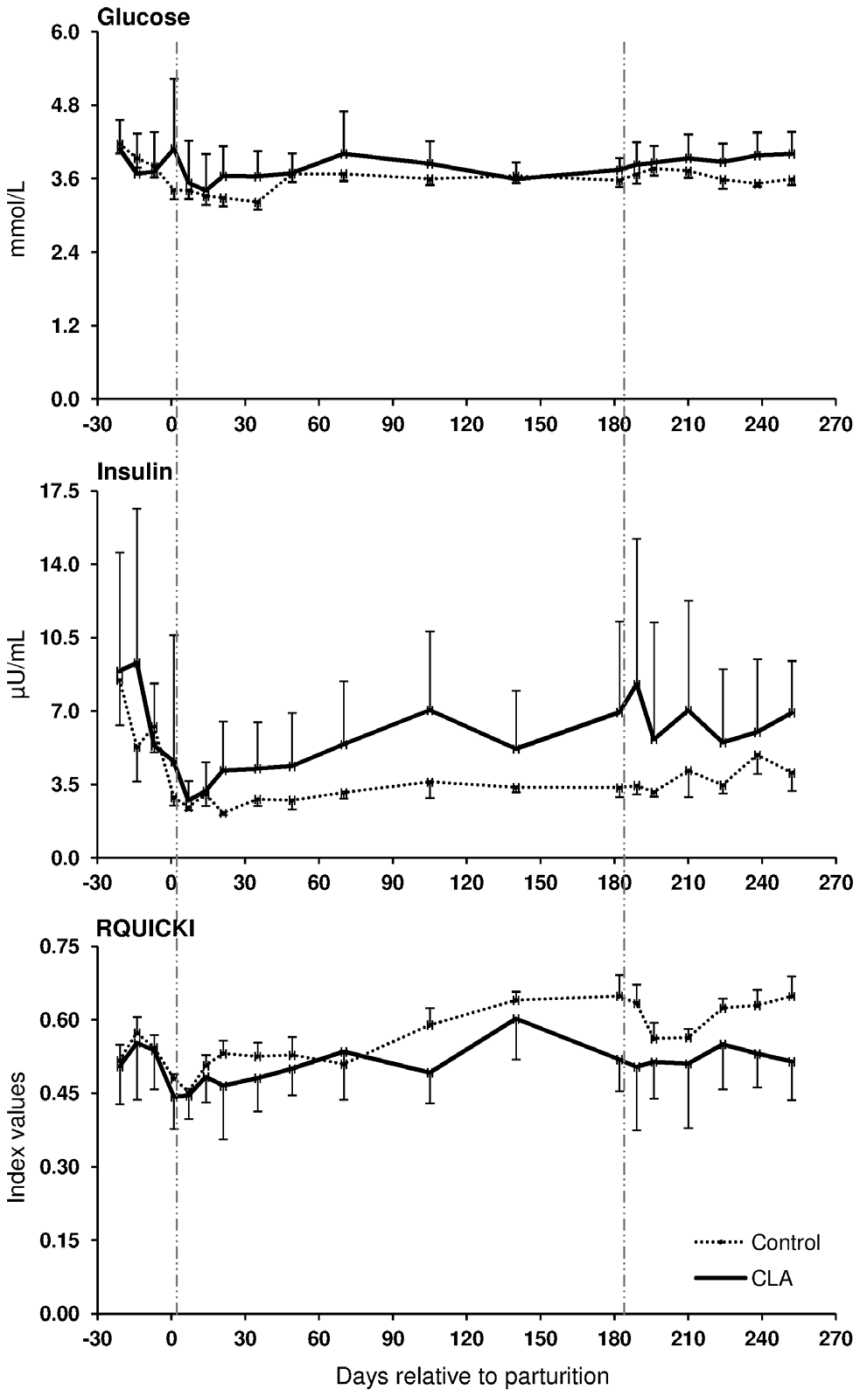
Conjugated linoleic acids effects on systemic insulin sensitivity in primiparous vs. pluriparous cows during lactation. Glucose and insulin concentration in serum and calculated Revised Quantitative Insulin Sensitivity Check Index (RQUICKI) in cows receiving conjugated linoleic acids (CLA, Lutrell® Pure, BASF SE, Ludwigshafen, Germany) at 100 g/day or a control fat supplement (Silafat®, BASF SE) from day 1 until day 182 postpartum [pluriparous cows (Control, n = 10 and CLA, n = 11) and primiparous cows (Control, n = 4 and CLA, n = 5)] in Trial 1. RQUICKI  = 1/[log(glucose) + log(insulin) + log(NEFA)], [26]. Supplementation with CLA did not interfere with the parameters measured in primiparous cows. Area between vertical lines corresponds to the CLA supplementation period (means ± SEM).

The coefficients of correlation calculated between the mRNA abundance of the target genes and blood glucose, insulin, NEFA and BHBA, RQICKI as well as with back fat thickness, body condition score and live weight (Table 2) ranged between r = 0.440 (LEP mRNA in s.c. AT and insulin) and r = 0.180 (LEPRB mRNA in liver and NEFA). Most correlations were related to ADIPOR1 mRNA in s.c. AT (7 out of 8 variables) which were negative for NEFA and BHB but there was no relation with back fat thickness. In contrast, significant correlations of ADIPOR2 mRNA in s.c. AT were limited to body condition score (BCS) and liver weight, whereas ADIPOR2 mRNA in liver had more correlations (6 out of 8 variables). These correlations were negative for glucose and insulin and liver weight but positive for NEFA and BHB. Serum LEP correlated positively with back fat thickness, comparable with LEP mRNA in s.c. AT (r = 0.225 vs. r = 0.245). LEPR in s.c. AT was positively associated with back fat thickness, but LEPR and LEPRB mRNA in liver were negatively correlated with back fat thickness. PPARγ mRNA in liver was negatively correlated with NEFA, BHB, insulin and back fat thickness but positively associated with the RQUICKI index. Supplementation with CLA only sporadically increased or decreased the expression of few genes at discrete time points in liver and s.c. AT; however, no consistent changes were observed. Treatment with CLA increased the mRNA abundance of LEPRB and TNF-α mRNA abundance in liver, whereas PPARγ mRNA was lower in CLA cows than in the controls at defined time points throughout lactation (Figure 3). Lower values than in the control group were also observed at 196 DIM i.e. beyond the supplementation period in case of PPARγ2 in s.c. AT of CLA cows (Figure 3). When testing CLA effects on insulin, glucose, and NEFA, and RQUICKI values, CLA effects were evident: the insulin concentrations were greater and RQUICKI data were lower (8% or 17% during supplementation and depletion period, respectively) than in the control group (Figure 4).

**Table 2:** Coefficients of correlation* between the mRNA abundance of the genes of interest and of serum leptin with blood analyses and variables of body condition in pluriparous cows.

Gene/Protein	Sample	Glucose [mmol/L]	Insulin [µU/ml]	^11^NEFA [mmol/L]	^12^BHBA [mmol/L]	^13^RQUICKI	Back fat	^14^BCS	Live weight
1LEP protein	serum	ns	ns	ns	ns	ns	.225**	ns	ns
1 *LEP*	s.c. fat	.241*	.440**	−.268**	ns	ns	.245*	.355**	.328**
^2^ *LEPR*	s.c. fat	ns	ns	.260**	ns	−.293**	.408**	.323**	−.260*
^2^ *LEPR*	liver	−.353**	−.426**	.266**	.190*	ns	−.351**	−.331**	ns
^3^ *LEPRB*	liver	ns	ns	.180*	ns	ns	−.275**	−.230**	ns
^4^ *ADIPOQ*	s.c. fat	ns	ns	ns	ns	ns	.226**	.282**	ns
^5^ *ADIPOR1*	s.c. fat	.201*	.194*	−.365**	−.246**	.184*	ns	.226*	.214*
^6^ *ADIPOR2*	s.c. fat	ns	ns	−.195*	ns	ns	ns	.196*	.424**
^5^ *ADIPOR1*	liver	ns	ns	ns	ns	ns	ns	−.179*	.244**
^6^ *ADIPOR2*	liver	−.343**	−.235**	.509**	.283**	−.170*	ns	ns	−.232*
^7^ *PPARγ*	s.c. fat	ns	.194*	−.191*	ns	ns	.244**	.247**	ns
^8^ *PPARγ2*	s.c. fat	ns	ns	ns	ns	ns	.384**	.377**	ns
^7^ *PPARγ*	liver	ns	−.176*	−.219**	−.241**	.288**	−.236**	ns	ns
^9^ *IL-6*	liver	ns	ns	ns	ns	ns	.349**	.228**	ns
^10^TNF-α	s.c. fat	ns	ns	.301**	ns	ns	ns	ns	ns

Person's product-moment correlation from merged data of the control and the CLA group throughout lactation. Significant correlations are indicated by * (*P*≤0.05) and ** (*P*≤0.01). ns: not significant.

^1^ Leptin (unit for serum LEP = ng/mL), ^2^Leptin receptor, ^3^Leptin receptor isoform b, ^4^Adiponectin, ^5^Adiponectin receptor 1, ^6^Adiponectin receptor 2, ^7^Peroxisome proliferator-activated receptor *gamma*,^ 8^Peroxisome proliferator-activated receptor *gamma2*, ^9^ Interleukin-6, ^10^Tumor necrosis factor-α, ^11^Non esterified fatty acids, ^12^Beta-hydroxy butyric acid, ^13^Revised Quantitative Insulin Sensitivity Check Index, ^14^Body Condition Score.

### Trial 2

Comparing the temporal changes of the different target genes across the first 105 days of lactation in different adipose depots, liver, muscle, and mammary gland of primiparous cows (Table 3 and 4), the most changes with time were observed for ADIPOR1. In mammary gland and all fat depots except omental AT, the receptor mRNA abundance increased with DIM. In contrast, the amount of this mRNA was reduced in muscle at d 42 and d 105, and at d 42 in liver tissue when compared against d 1. The mRNA abundance of ADIPOR2 increased with DIM in retroperitoneal AT; in mammary gland the values were higher at d 105 compared to d 42. The ADIPOQ mRNA abundance increased in two v.c. AT (omental and retroperitoneal) with DIM. LEPRB mRNA abundance was decreased with DIM in all v.c. AT and in the mammary gland. No changes were found for LEP mRNA in primiparous cows during lactation. The mRNA abundance for PPARγ and PPARγ2 changed in some tissues but not consistently. For PPARγ an increase over time was detected in omental AT, and for PPARγ2 in retroperitoneal AT. In addition, an increase was observed in liver for both transcripts. The amount of TNF-α mRNA increased with DIM in all s.c. AT and in liver; in mammary gland; in retroperitoneal AT, a decrease with DIM was observed. The mRNA of IL-6 was decreased in s.c. AT from tail head and withers but increased in liver over the time.

**Table 3:** *ADIPOQ* system and Peroxisome proliferator-activated receptor γ and γ2 mRNA expression in different tissues of primiparous cows supplemented with or without CLA in a time course of 105 days in milk (DIM).

Gene	DIM	Treatment	Tissue mRNA abundance (arbitrary unit)								
			Omental	Mesenteric	Retroperitoneal	s.c. tail head	s.c. withers	s.c. sternum	Muscle^1^	Mammary^2^	Liver
*ADIPOQ* ^3^	1	Control	0.63±0.20^a^	0.69±0.31	1.04±0.24^a^	1.16±0.15	1.62±0.52	0.87±0.23		0.10±0.02	
	42	Control	0.89±0.36^a^	0.41±0.12	1.05±0.20^a^	1.24±0.28	1.08±0.68	0.68±0.21		0.16±0.05	
	42	CLA	1.35±0.41	0.62±0.37	1.22±0.40^a^	0.90±0.63	1.13±0.48	0.61±0.21		0.42±0.26	
	105	Control	**2.11**±**0.51** ^b^	1.14±0.58	2.86±0.49^b^	1.91±0.39	1.16±0.47	0.53±0.27		0.20±0.04	
	105	CLA	**0.83**±**0.44**	0.88±0.35	1.71±0.46^b^	1.22±0.28	1.66±0.38	1.09±0.26		0.30±0.09	
*ADIPOR1* ^4^	1	Control	0.80±0.05	0.60±0.06^a^	0.80±0.09^ab^	0.85±0.04^a^	0.89±0.06^a^	0.84±0.05^a^	1.69±0.14^a^	0.89±0.05^a^	0.87±0.06
	42	Control	0.87±0.18	0.62±0.03^a^	0.88±0.06^a^	0.89±0.14^a^	0.80±0.13	0.84±0.06^a^	0.94±0.06^b^	0.91±0.07^a^	0.74±0.05^a^
	42	CLA	1.08±0.12	0.67±0.05^a^	0.79±0.04^a^	0.79±0.12	1.35±0.26	0.83±0.08^a^	1.06±0.10^b^	0.92±0.11^a^	0.75±0.07^a^
	105	Control	1.07±0.12	0.88±0.15^b^	1.18±0.11^b^	1.34±0.16^b^	1.28±0.17^b^	1.07±0.09^b^	1.06±0.05^b^	1.15±0.08^b^	0.87±0.03^b^
	105	CLA	0.85±0.18	0.76±0.05^b^	1.03±0.13^b^	1.06±0.14	1.28±0.15^b^	1.20±0.13^b^	0.93±0.05^b^	1.15±0.05^b^	0.88±0.04^b^
*ADIPOR2* ^5^	1	Control	1.70±0.60	1.97±0.70	3.65±0.80	3.23±0.76	4.51±1.64	2.64±0.90	1.35±0.08	1.43±0.53	1.62±0.39
	42	Control	1.87±0.86	1.39±0.40	3.22±0.42^a^	3.01±1.04	2.87±2.55	1.81±0.82	1.19±0.18	0.77±0.17^a^	1.44±0.32
	42	CLA	3.03±1.24	1.92±1.03	3.21±1.04^a^	2.68±2.04	2.88±1.38	2.32±0.86	0.99±0.08	0.95±0.30^a^	1.56±0.30
	105	Control	3.68±1.32	2.21±1.01	**5.94**±**1.31** ^b^	5.21±1.48	3.69±2.06	1.98±1.15	1.42±0.10	2.56±0.61^b^	1.29±0.15
	105	CLA	2.02±1.02	1.37±0.57	**2.56**±**0.70** ^b^	2.18±0.71	2.56±1.23	2.81±1.18	1.22±0.11	1.86±0.16^b^	1.06±0.05
*PPARγ* ^6^	1	Control	1.18±0.13^a^	1.58±0.27	1.76±0.30	1.97±0.32	2.34±0.26	1.57±0.28			0.65±0.11^a^
	42	Control	1.50±0.45^ab^	1.57±0.28	2.94±0.35	2.45±0.50	1.57±0.48	1.74±0.17			0.78±0.05^ab^
	42	CLA	2.04±0.34^ab^	1.92±0.24	2.45±0.30	1.59±0.22	1.82±0.42	1.29±0.34			0.76±0.07^ab^
	105	Control	1.98±0.45^b^	2.48±0.42	3.57±0.49	2.56±0.47	2.05±0.51	1.67±0.27			0.85±0.11^b^
	105	CLA	2.47±0.45^b^	3.25±0.66	3.32±0.78	2.53±0.53	2.99±0.52	2.34±0.38			1.10±0.12^b^
*PPARγ2* ^7^	1	Control	0.88±0.15	0.87±0.25	0.92±0.25^a^	1.47±0.16	1.78±0.45	0.83±0.23	0.69±0.13	1.15±0.39	0.56±0.06^a^
	42	Control	1.42±0.47	0.76±0.09	1.33±0.21^ab^	1.71±0.49	0.80±0.39	0.91±0.16	0.82±0.04	**1.15**±**0.23**	0.68±0.08^a^
	42	CLA	1.76±0.32	1.14±0.25	1.19±0.06^ab^	1.13±0.31	1.17±0.31	0.62±0.14	0.82±0.16	**0.68**±**0.05**	0.65±0.08^a^
	105	Control	1.47±0.37	1.29±0.23	1.59±0.13^b^	1.59±0.23	0.97±0.26	0.74±0.06	0.71±0.09	**0.97**±**0.14**	0.98±0.25^b^
	105	CLA	1.24±0.33	1.11±0.20	1.45±0.24^b^	1.55±0.36	1.57±0.21	1.13±0.15	0.78±0.12	**0.75**±**0.12**	1.23±0.21^b^

CLA: Lutrell® Pure, BASF SE, Ludwigshafen, Germany.

Control: Silafat®, BASF SE.

Significant differences (*P*<0.05) between different days per tissue are defined using different letters. Significant differences (*P*≤0.05) between CLA and control group within day and tissue are depicted by bold numbers.

Data are normalized based on the geometric mean of Eukariotic translation initiation factor 3 (EIF3K), Lipoprotein receptor-related protein 10 (LRP10), RNA polymerase II (POLR2A), Emerin (EMD), Marvel domain containing 1 (MARVELD1), and Hippocalcin-like 1 (HPCAL1) for each s.c. fat and mesenteric fat depots; EIF3K, LRP10, POLR2A, EMD, and MARVELD1 for omental and retroperitoneal fat depots; HPCAL1, LRP10, POLR2A, EIF3K, Glyceraldehyde-phosphate-dehydrogenase (GAPDH) for liver; LRP10, EMD, POLR2A, EIF3K for muscle, and MARVELD1, EMD, LRP10, EIF3K, POLR2A, HPCAL1 for mammary gland tissue.

^1^ Semitendinosus. ^2^ *ADIPOQ* in mammary gland tissue was only detectable in one-third of the samples. ^3^ Adiponectin. ^4^ Adiponectin receptor 1. ^5^ Adiponectin receptor 2. ^6^ Peroxisome proliferator-activated receptor γ. ^7^ Peroxisome proliferator-activated receptor γ2; Means ± SE.

**Table 4:** *LEP* system, *IL-6*, and *TNF-α* mRNA expression in different tissues of primiparous cows supplemented with or without CLA in a time course of 105 DIM.

Gene	DIM	Treatment	Tissue mRNA abundance (arbitrary unit)								
			Omental	Mesenteric	Retroperitoneal	s.c. tail head	s.c. withers	s.c. sternum	Muscle	Mammary	Liver
*LEP* 1	1	Control	0.43±0.11	0.80±0.27	1.70±0.37	0.87±0.21	1.14±0.34	0.77±0.27	0.18±0.09	0.29±0.15	
	42	Control	0.89±0.38	0.67±0.25	1.07±0.38	1.01±0.39	0.73±0.67	1.24±0.60	0.18±0.08	0.35±0.10	
	42	CLA	1.67±0.57	0.88±0.30	1.44±0.55	0.92±0.61	1.85±1.10	1.63±0.83	0.71±0.28	0.43±0.17	
	105	Control	2.23±1.11	1.33±0.67	3.33±0.65	1.40±0.27	1.69±0.74	0.76±0.15	0.50±0.27	0.15±0.05	
	105	CLA	1.38±0.76	0.86±0.26	1.97±0.67	1.16±0.39	1.80±0.54	1.76±0.59	0.34±0.13	0.23±0.10	
*LEPRB* ^2^	1	Control	1.57±0.41^a^	0.67±0.19	1.60±1.06^ab^	0.54±0.14	1.19±0.49	2.42±0.85	0.35±0.05	0.52±0.07^a^	1.49±0.27
	42	Control	0.41±0.12^b^	0.67±0.20^a^	1.35±0.31^a^	0.42±0.30	0.72±0.32	2.45±1.10	0.71±0.14	0.26±0.11^ab^	1.29±0.14
	42	CLA	0.51±0.20^b^	0.79±0.26^a^	1.57±0.53^a^	0.22±0.05	1.06±0.21	0.98±0.15	0.70±0.11	0.37±0.03^ab^	1.98±0.41
	105	Control	0.97±0.67^b^	0.27±0.11^b^	0.54±0.17^b^	0.27±0.10	0.44±0.14	1.14±0.23	0.59±0.10	0.17±0.11^b^	1.20±0.18
	105	CLA	0.56±0.18^b^	0.34±0.16^b^	0.58±0.21^b^	0.24±0.09	1.06±0.45	2.33±0.84	0.72±0.14	0.28±0.08^b^	1.37±0.16
*TNF-α* ^3^	1	Control	1.14±0.18	0.58±0.10	1.06±0.16	0.65±0.05^a^	0.58±0.09^a^	1.08±0.19^a^	0.72±0.13	1.81±0.33^a^	0.52±0.06^a^
	42	Control	1.37±0.40	**1.00**±**0.10**	0.92±0.12^a^	1.27±0.21^b^	1.37±0.28^b^	1.45±0.36^ab^	1.03±0.16	0.81±0.14^b^	1.12±0.20^b^
	42	CLA	1.33±0.36	**0.72**±**0.25**	1.09±0.17^a^	1.74±0.63^b^	1.06±0.22^b^	1.34±0.23^ab^	1.08±0.38	0.96±0.12^b^	1.42±0.11^b^
	105	Control	0.83±0.27	0.84±0.11	0.64±0.16^b^	1.03±0.37^ab^	1.13±0.39^ab^	2.25±0.53^b^	0.92±0.12	0.99±0.14^b^	1.62±0.21^b^
	105	CLA	1.70±0.43	1.12±0.37	0.69±0.11^b^	1.15±0.27^ab^	1.01±0.12^ab^	1.77±0.39^b^	0.94±0.12	0.68±0.13^b^	1.56±0.27^b^
*IL-6* ^4^	1	Control	2.77±1.57	0.85±0.41	1.43±0.36	4.30±2.29^a^	7.31±2.07^a^	0.52±0.13	0.85±0.20	0.68±0.14	0.36±0.08^a^
	42	Control	0.82±0.09	0.68±0.08	0.88±0.31	1.62±0.40^ab^	2.79±1.38^b^	0.53±0.13	0.88±0.18	0.83±0.10	0.70±0.10^b^
	42	CLA	0.74±0.06	0.79±0.31	0.70±0.07	1.38±0.55^ab^	1.68±0.36^b^	0.34±0.13	0.86±0.13	0.83±0.19	0.78±0.15^b^
	105	Control	1.08±0.66	0.36±0.15	0.59±0.15	0.90±0.25^b^	0.69±0.36^b^	0.26±0.09	0.76±0.11	0.95±0.14	0.88±0.11^b^
	105	CLA	3.98±1.37	1.65±0.16	2.13±1.41	3.65±2.53^ab^	2.22±0.73^b^	0.55±0.15	0.94±0.14	0.82±0.09	0.73±0.12^b^

CLA: Lutrell® Pure, BASF SE, Ludwigshafen, Germany.

Control: Silafat®, BASF SE.

Significant differences between different days per tissue are defined using different letters. Significant differences (P≤0.05) between CLA and control group within day and tissue are depicted by bold numbers.

Data are normalized based on the geometric mean of Eukariotic translation initiation factor 3 (EIF3K), Lipoprotein receptor-related protein 10 (LRP10), RNA polymerase II (POLR2A), Emerin (EMD), Marvel domain containing 1 (MARVELD1), and Hippocalcin-like 1 (HPCAL1) for each s.c. fat and mesenteric fat depots; EIF3K, LRP10, POLR2A, EMD, and MARVELD1 for omental and retroperitoneal fat depots; HPCAL1, LRP10, POLR2A, EIF3K, Glyceraldehyde-phosphate-dehydrogenase (GAPDH) for liver; LRP10, EMD, POLR2A, EIF3K for muscle, and MARVELD1, EMD, LRP10, EIF3K, POLR2A, HPCAL1 for mammary gland tissue.

^1^ Leptin. ^2^ Leptin receptor isoform b. ^3^ Tumor necrosis factor-α. ^4^ Interleukin-6; Means ± SE.

Summarising the associations between blood metabolites, body fat and liver mass with ADIPOQ mRNA abundance in different AT depots, negative correlations were observed between ADIPPOQ mRNA in the mesenteric AT depot and omental and mesenteric fat mass (r = −0.433 and r = −0.484, respectively) as well as with total fat mass (i.e. mass of s.c. and all v.c. depots taken together; r = −0.432). In addition, ADIPOQ mRNA in retroperitoneal AT was negatively correlated with NEFA (r = −0.428).

Correlation analysis between the mRNAs of ADIPOR1 and ADIPOR2 in different AT depots and adipose tissue masses as well as liver mass revealed negative correlations between ADIPOR1 in mesenteric AT and AT masses (omental: r = −0.462, mesenteric: r = −0.461, retroperitoneal: r = −0.467, mass of s.c. fat and all v.c. depots: r = −0.480), but a positive correlation with liver mass was observed for ADIPOR1 in two v.c. depots (mesenteric: r = 0.407 and retroperitoneal: r = 0.456) and in the s.c. depot from sternum (r = 0.633). For ADIPOR2 correlations were limited to the s.c. depots, mainly the one from the withers area: positive associations were established with omental (r = 0.479), retroperitoneal (r = 0.491), total s.c. AT mass and total AT mass of all depots considered (r = 0.426 and r = 0.494).

Supplementation with CLA only had few and inconsistent effects on gene expression in primiparous cows (Table 3 and 4). The mRNA abundance of TNF-α mRNA was reduced by CLA at 42 DIM in mesenteric AT, and the ADIPOQ and ADIPOR2 mRNA abundance were decreased at 105 DIM in omental and retroperitoneal AT, respectively. The mRNA abundance of PPARγ2 was reduced by CLA only in the mammary gland at both 42 and 105 DIM.

### Differences in gene expression of target genes between individual adipose tissue depots and in other tissues

**Figure 5:**
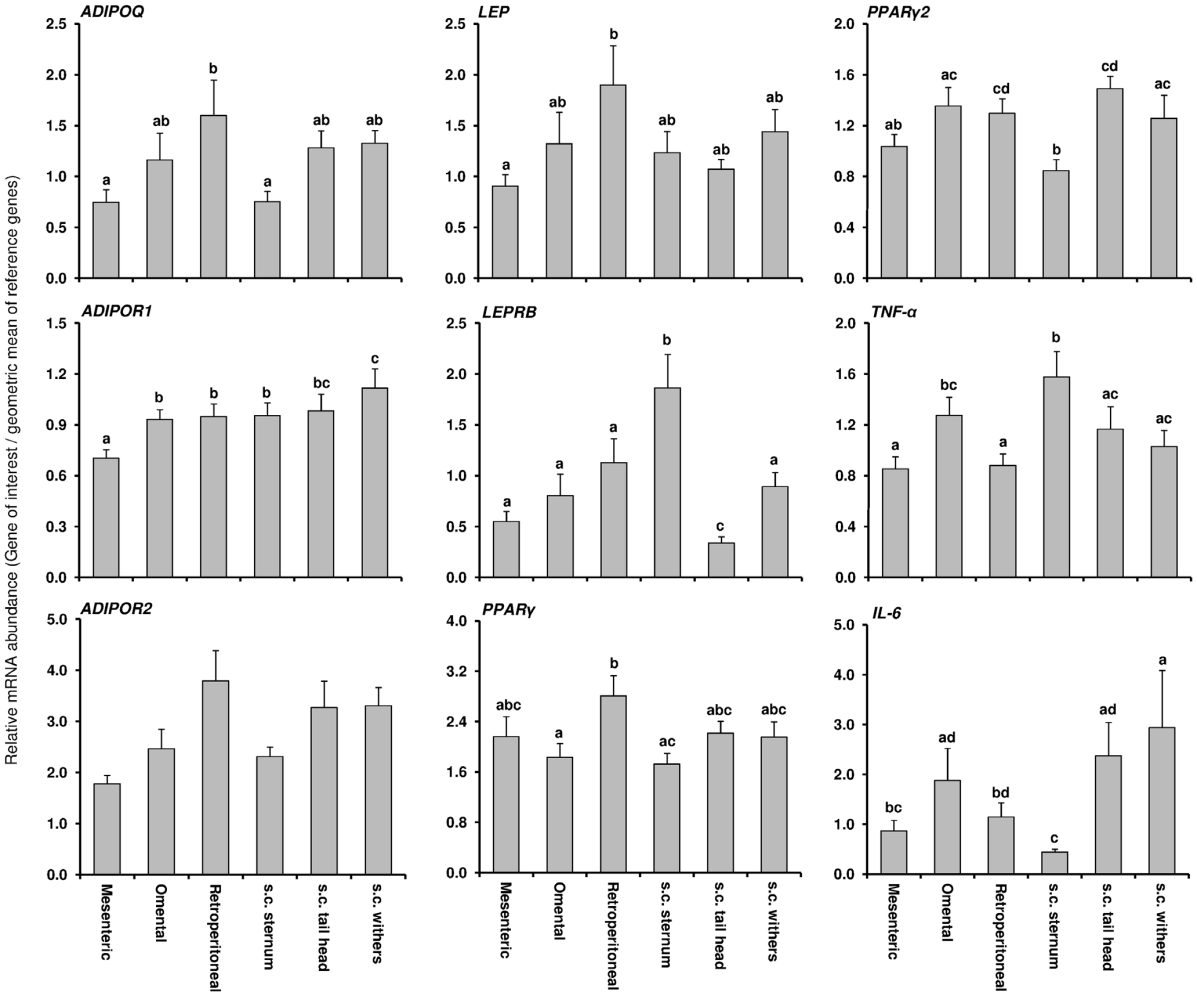
Average mRNA expression of genes related to insulin sensitivity in six different fat depots from primiparous cows. Means (± SEM) are presented across sampling times and treatments for adiponectin (ADIPOQ), adiponectin receptors (ADIPOR1 and ADIPOR2), leptin (LEP), leptin receptor isoform b (LEPRB), peroxisome proliferator-activated receptor (PPAR) *γ* and *γ2*, tumor necrosis factor (TNF)*-α*, and interleukin (IL)*-6* mRNA abundance. Different letters specify the differences between individual fat depots (*P*≤0.05). Data were normalized based on the geometric mean of eukariotic translation initiation factor 3 (EIF3K), lipoprotein receptor-related protein 10 (LRP10), RNA polymerase II (POLR2A), emerin (EMD), marvel domain containing 1 (MARVELD1), and hippocalcin-like 1 (HPCAL1).

The mRNA abundance of the target genes was compared between different AT from Trial 2 irrespective of treatment and DIM (Figure 5). Differences between the different AT depots were seen for all target mRNAs except ADIPOR2. In addition, the mRNA abundance of most target genes was different when comparing only within v.c. depots or within s.c. depots.

By the real-time PCR protocol applied, ADIPOQ mRNA was detectable in the mammary gland only in one third of the samples. The abundance was about 900 times lower than in AT. For ADIPOR1, a 2 fold lower abundance was observed in the liver than in the average of all AT whereas in muscle the values were 3 fold higher than in AT. The abundance of ADIPOR2 mRNA in muscle was higher than in liver. In both tissues the abundance was mostly in the same range as in the different AT depots although liver and muscle had lower and higher ADIPOR2 mRNA abundance in comparison to retroperitoneal and mesenteric AT, respectively. Compared to AT, there was about 100 fold less PPARγ2 mRNA in liver, 79 fold less in muscle and 17 times less in mammary gland. PPARγ mRNA abundance in liver was 109 fold lower in comparison to the mean of AT. LEP mRNA abundance was low in muscle and mammary gland in comparison to the mean of fat depots (171 and 353 fold lower, respectively). In liver, 34 times more LEPRB mRNA abundance than in AT was observed. About 5 fold more TNF-α mRNA was observed in liver than in AT, but IL-6 mRNA abundance was 8.3 times lower in liver than in AT.

## Discussion

### Gene expression of the ADIPOQ and LEP system during lactation

In general, the adipokines LEP and ADIPOQ are considered as positive regulators of IS as demonstrated in monogastrics [4]. Skeletal muscle lipid stores are linked to insulin secretion and peripheral insulin sensitivity. LEP improves insulin sensitivity by increasing the activity of AMP-activated protein kinase (AMPK) in muscle which reduces the activity of acetyl-CoA carboxylase, therewith reducing lipid stores by increasing fatty acid oxidation in skeletal muscle [36]. On the other hand it should be taken into account that LEP also increases pro-inflammatory protein secretion like TNF-α by macrophages [37] but reduces pro-inflammatory protein secretion indirectly by activation of AMPK e.g. in adipose tissue [38]. In contrast to LEP, ADIPOQ acts anti-inflammatory e.g. by reducing TNF-α secretion. A main target of ADIPOQ is AMPK. Its activation in liver is associated with reduced gluconeogenesis by the reduction of phosphoenolpyruvate carboxykinase and glucose-6-phosphatase expression and increased fatty acid oxidation by inactivation of acetyl-CoA carboxylase [39]. LEP is positively and ADIPOQ is negatively linked with body fat content [6], [7]. Effects of LEP are mainly mediated by LEPRB [8] and ADIPOQ via ADIPOR1 and ADIPOR2, as observed in humans [9]. As confirmed herein, the blood concentrations of LEP in dairy cows are low in early lactation [40] and thus may affect the secretion of pro-inflammatory proteins as discussed above. Correlation analyses revealed deviant regulation of LEPR and LEPRB mRNA between back fat thickness and BCS in s.c. AT (positively correlated) vs. liver (negatively correlated). In liver LEPR mRNA was also negatively correlated with glucose and insulin but positively with NEFA and BHB. This might be an indication of the importance of LEP in regulating liver lipid content and gluconeogenesis [41]. In type 2 diabetic mice LEP improves hepatic insulin sensitivity by reducing gluconeogenesis which should be associated with the negative correlations between LEPR mRNA vs. glucose and insulin. On the other hand, possibly more relevant for dairy cows, LEP increases fatty acid oxidation in liver and reduces hepatic lipotoxicity [41], and decreased LEP at parturition might contribute to the increased vulnerability to hepatic steatosis. The observed upregulation of the receptor mRNA for LEPR and LEPRB in liver as well as the positive correlation with NEFA and in case of LEPR mRNA also with BHBA indicates a counterbalancing of LEP effects at the receptor level in this situation.

For ADIPOQ less information is available in cattle but decreased concentrations were documented using semiquantitative Western blots [42], [43] and a bovine specific ELISA [43].

Koltes and Spurlock [44] observed a decrease of the ADIPOQ mRNA abundance in s.c. AT from tail head throughout the transition period. This result is comparable to our observation in Trial 1 in pluriparous cows and may affect signalling by ADIPOQ receptors which were down regulated in s.c. fat after parturition. For the LEPR mRNA in s.c. AT, our present results are not in line with data published by our group and others [45], [46]. In these two studies, an increase in LEPRB and LEPR mRNA, respectively was observed in s.c. AT and explained by Thorn et al. [46] at least partly by the low insulin concentrations during early lactation. As expected, we also observed low insulin concentrations after calving.

In humans, ADIPOQ and its receptors are inversely related to body fat mass; both receptors and serum ADIPOQ are down-regulated by insulin in lean men [9], [47]. The reduction in mRNA abundance of the ADIPOQ system we observed during the transition period in s.c. AT of pluriparous cows in Trial 1 therefore indicates an uncoupling between energy balance and the ADIPOQ system as they were not regulated contrary to the energy status and insulin values as shown in humans and mice [9], [47]. ADIPOQ mRNA was positively correlated with back fat thickness and BCS, as well as ADIPOR1/2 mRNA in s.c. AT thus emphasising the differential regulation in cows as compared to humans or mice at least at the mRNA level. Based on the number of correlations between the analysed parameters and ADIPOR1 mRNA in s.c. AT of pluriparous cows this receptor might be more relevant for paracrine/autocrine ADIPOQ effects in this AT depot than ADIPOR2; the latter seems to be closer linked to liver of pluriparous dairy cows since ADIPOR1 was positively but ADIPOR2 mRNA was negatively correlated with the mass of this organ. ADIPOR2 mRNA is more abundant than ADIPOR1 mRNA in liver of cattle [48], which may underline its importance in fatty acid oxidation by PPARα signalling [49]. ADIPOQ is associated with lipogenesis and differentiation during adipogenesis at least in murine 3T3-L1 adipocytes [50] and we observed a positive correlation with back fat thickness and BCS in pluriparous cows. The decreased ADIPOQ mRNA abundance in AT during early lactation might therefore be related to reduced lipogenesis and adipogenesis during NEB in discrete AT depots. The regulation of both receptors in liver differs from s.c. AT as the ADIPOR1 mRNA abundance was stable and the one for ADIPOR2 increased in liver during the transition period. Differential regulation of both receptors in these two tissues is underlined by different correlation patterns of both receptor mRNAs in s.c. AT and liver. More correlations with the different variables (e.g. negative correlations with NEFA and BHBA) were found for ADIPOR1 in s.c. AT and for ADIPOR2 (e.g. negative correlation with glucose and insulin, positive correlation with NEFA) in liver. Signalling by ADIPOR2 in liver during negative energy balance may underline a positive link to fatty acid oxidation and energy metabolism in cattle as suggested for mice [49]. Based on ADIPOQ signal transduction in liver as discovered in a mouse model with targeted disruption of the ADIPORs [49], reduced ADIPOQ serum concentrations during the transition period may enhance gluconeogenesis. The mRNA of ADIPOR2 increased after parturition in liver of pluriparous cows in this study. Similar observations were reported by Loor et al. [51]. In their study the expression of the receptor mRNA was further increased by feed restriction before calving. In addition, growth hormone (GH) might be related to this observation: GH knowingly peaks around parturition in dairy cattle [52] and ADIPOR2 but not ADIPOR1 was shown to be upregulated by GH in liver of mice [53]. Signalling by ADIPOR2 is associated with less oxidative stress in liver [49], and oxidative stress is increased by NEFA as shown in human hepatocytes [54]. During NEB in early lactation, NEFA concentrations increase [3]. Based on the fact that targeted ADIPOR2 disruption in liver of mice increased oxidative stress markers [49], upregulation of ADIPOR2 may help to mitigate oxidative stress in liver of dairy cows, that is increased due to lipid mobilization in the periparturient period [55].

In the primiparous cows from Trial 2, most of the temporal changes occurred for ADIPOR1 mRNA in several adipose depots, in particular in the s.c. AT depots in which the abundance increased with DIM. As mentioned earlier, this might also be related to the effects of ADIPOQ on adipogenesis and lipogenesis [50] which are down-regulated during NEB with increased lipolysis [56]. As discussed by Dagon et al. [57] and Rossmeisl et al. [58], AMPK is an important mediator of hormonal and nutritional effects on AT and is thus related to the control of body fat mass. Its activation involves the down-regulation of lipogenic and adipogenic genes like PPARγ. Both ADIPORs are linked to AMPK activation but ADIPOR1 to a higher extent than ADIPOR2, being closer associated with PPARα signalling, at least in liver [49]. Therefore, an increase in abundance of ADIPOR1 until peak lactation might be related to an increase in AMPK activity, controlling lipolysis tightly in retroperitoneal AT and in different s.c. AT of primiparous cows. Based on a recent study, the energy sensor AMPK may inhibit lipolysis and hormone sensitive lipase transport to the lipid droplet [59], which is in line with our observation of increasing ADIPOR1 mRNA abundance in AT. Therefore, AMPK may protect the adipocyte against energy depletion as discussed by Gauthier et al. [60]. Antilipolytic effects of AMPK were also addressed for dairy cows based on increasing phosphorylation of AMPKα1 after parturition [61]. Summarising, increasing ADIPOR1 mRNA throughout lactation might be associated with reduced lipolysis in dairy cows by increasing AMPK activity which would be in line with positive effects of ADIPOQ on lipid accumulation in AT [50] and lowest ADIPOQ concentrations after parturition in dairy cows [43]. Interestingly, in the primiparous cows we observed negative correlations between mesenteric ADIPOQ mRNA and the omental and mesenteric AT mass comparable to ADIPOR1 mRNA in this depot which was also negatively correlated with retroperitoneal AT mass as well as total AT mass. This observation could be related to the stage of lactation (sampling dates: DIM 1, 42, 105) and negative ADIPOQ effects on gluconeogenesis [39] as well as the cows' performance level. AT from cows at DIM 100 of two families with differences in their capability to store body fat and to secrete milk revealed positive correlations between mesenteric ADIPOQ mRNA and omental as well as mesenteric AT mass [48]. In humans, mesenteric AT has an important role in regulating IS [62], and possibly also in cows. High AT mass of this depot might be unfavourable by its secretion products on gluconeogenesis and hepatic IS in early lactation of high yielding dairy cows.

### Gene expression of PPARγ and PPARγ2 during lactation

The transcription factor PPARγ is a key regulator of IS and adipogenesis [63]. Targeted deletion of PPARγ in murine liver decreased hepatic triglyceride content and impaired systemic IS [64]. Therefore, regulation of PPARγ around calving may affect the insulin responsiveness of tissues. Like in other species, two PPAR*γ* isoforms have been identified in cattle i.e. PPARγ1 and PPARγ2 [16]. In vitro, the isoform PPARγ2 has stronger adipogenic activity than the isoform PPARγ1 [65] and might at least partly explain the positive associations we found between PPARγ and PPARγ2 vs. back fat thickness and BCS.

In the pluriparous cows of Trial 1, PPARγ was reduced in s.c. AT and in the liver from late pregnancy to early lactation. We suggest that the expression patterns of PPARγ and PPARγ2 mRNA, with highest values prepartum compared to postpartum in AT, are possibly associated to the genes we analysed throughout lactation. Lower values in PPARγ mRNA abundance in liver were also reported 3 wk after parturition as compared to the first wk of lactation [66]. The nadir observed for PPARγ expression in AT but also in liver at wk 3 postpartum is in line with this report. In contrast, PPARα is upregulated after parturition [67]. As discussed by Lee et al. [68], the uppermost expression for PPARγ is found in AT, where it is related to adipogenesis and IS. In liver, it is linked to fatty acid uptake, lipid storage, and reduced gluconeogenesis. We observed negative correlations between PPARγ and NEFA, BHBA but, albeit weaker, also to insulin concentrations. This constellation in particular with regard to NEFA and BHBA may mitigate hepatic fatty acid uptake at least in pluriparous dairy cows. High abundance of PPARγ mRNA in s.c. AT during late pregnancy might be related to energy accretion by AT. In liver, the decreased abundance of PPARγ during early lactation may also result in increased gluconeogenic capacity.

In Trial 2, no changes in PPARγ and PPARγ2 were observed in s.c. AT after parturition in primiparous cows, which is in line with the results obtained in s.c. AT from Trial 1 with pluriparous cows. In addition to the potential effects of PPARγ on gluconeogenesis, the incidence for fatty liver might be reduced by decreasing expression of PPARγ2 after parturition: inactivation of PPARγ2 may improve fatty liver induced by high fat diet in mice [69]. This suggestion is in line with our observation about the negative correlations between PPARγ vs. NEFA and BHBA in pluriparous cows as discussed above.

For the mammary gland, Bionaz and Loor [70] reported that PPARγ is upregulated at the onset of lactation in comparison to 15 DIM. Comparing 1, 42 and 105 DIM in our study, PPARγ2 mRNA remained constant in mammary gland, similar to another study [71].

### Gene expression of the pro-inflammatory proteins IL-6 and TNF-α

The adipokines IL-6 and TNF-α are related to insulin resistance [72]. In general, TNF-α reduces IS by phosphorylation of insulin receptor substrate which in turn blocks insulin signalling [73]. In consequence, plasma NEFA concentrations rise thus further enhancing insulin receptor substrate phosphorylation indirectly [74]. TNF-α increases factors related to fatty acid uptake in liver but does not affect genes associated with fatty acid oxidation as demonstrated in TNF-α knockout mice [75]. Accordingly, the intracellular rise of NEFA in non-alcoholic fatty liver increases the hepatic diacylglycerol content, which further reduces IS as discussed above [76].

In dairy cows, plasma TNF-α peaks transiently at parturition and decreases thereafter below prepartum values [77]; high TNF-α serum concentrations are associated with insulin resistance and fatty liver 1 to 2 wk after calving [78]. For serum IL-6, no differences were observed at different stages of lactation [79]. Limited data is available about the mRNA expression of both adipokines in bovine AT, but liver TNF-α mRNA abundance is higher than the amount of IL-6 mRNA in liver [80].

In Trial 1, the TNF-α mRNA abundance remained fairly constant throughout lactation in liver and in s.c. AT. Besides confirming earlier reports focusing on the transition period [66], [81], we also provided data about TNF-α mRNA expression in later stages of lactation. In contrast to TNFα, IL-6 mRNA in liver decreased during the transition period. IL-6 not only has adverse effects on liver metabolism but also stimulates hepatocyte proliferation and prevents cell damage [82]. The observed decline towards early lactation might be related to a higher susceptibility for metabolic health disorders during early lactation [83]. The abundance of IL-6 mRNA in s.c. AT of pluriparous cows was too low for valid quantification by our protocol and was not detectable in each sample.

In primiparous cows (Trial 2) we observed an increase in TNF-α mRNA abundance in all s.c. AT, at least at one of the sampling dates after parturition, which is different from the studies of Sadri et al. [81] and van Dorland et al. [66] reporting no changes during early lactation in pluriparous cows. In retroperitoneal AT TNF-α mRNA was less abundant at 105 than on 1 DIM. In contrast to pluriparous cows we observed an increase of TNF-α mRNA abundance in liver. Therefore, we suggest that TNF-α mRNA expression differs between primiparous and pluriparous cows and between different AT of primiparous cows. Differences between the different AT depots might be related to local regulation of IS in either a paracrine or autocrine manner.

The IL-6 mRNA in s.c. AT from tail head and withers was reduced from 1 to 105 DIM. Therefore, the regulation of the pro-inflammatory proteins IL-6 and TNF-α seems different at least at the level of the mRNA in these AT depots. In contrast, the IL-6 mRNA in liver increased during this time. This observation might be related to an improved protective and regenerative capacity related to IL-6 at this stage of lactation [82] in primiparous, and, to a lower extent, in pluriparous cows, where only a numerical increase was observed. On the other hand, IL-6 is associated with reduced IS [72]. Network analysis in cows revealed that IL-6 is related to many liver specific pathways e.g. lipoprotein metabolism and fatty acid oxidation and increases during ketosis [84]. Therefore, lower abundance in liver around parturition and early lactation might be advantageous in relation to IS but the protection of the liver against oxidative stress during this time period might be reduced as mentioned previously.

As discussed by Alluwaimi [85], TNF-α is suggested to maintain and regulate immunological functions within the mammary gland and the decrease from 1 to 42 or 105 DIM, respectively, may reflect the adaptation of the gland to galactopoesis as compared to colostrogenesis and lactogenesis.

### Effects of CLA supplementation on gene expression

In general the effects of CLA supplementation were scarce and not consistent between genes, tissues and time points throughout lactation. However, in case of differences between control and CLA cows, the abundance of the respective target mRNA was always decreased, with only one exception (LEPRB in liver of pluriparous cows). CLA reduced ADIPOQ mRNA in omental and ADIPOR2 mRNA in retroperitoneal AT at 105 DIM. In addition, lower TNF-α mRNA abundance was observed in mesenteric AT of the CLA group, although only at 42 DIM. Comparable to a study of Sigl et al. [86] using primiparous Brown Swiss cows, we detected no CLA effect on hepatic PPARγ mRNA abundance in primiparous German Holstein cows, whereas CLA treatment in pluriparous cows resulted in sporadically decreased hepatic PPARγ mRNA abundance. CLA are ligands for PPARs [19], [20]. Nevertheless, the effects of CLA in primiparous dairy cattle may differ from the effects of the PPARγ agonist 2,4-thiazolidinedione observed in bulls [87]. However, the effects of 2,4-thiazolidinedione were not consistent in bulls comparable to our CLA effects. Therefore, inconsistency for PPARγ agonists like 2,4-thiazolidinedione and CLA between different tissues or AT depots in cattle may exists. This result is in contrast to the situation in mice, were broad consistency was observed regarding to the effects of both compounds on glucose and lipid metabolism [88].

We suppose that the impact of CLA supplementation is more important for the mammary gland than for all other analysed tissues. Treatment with CLA decreased PPARγ2 mRNA at both time points investigated in primiparous cows, accounting for 2 out of 5 CLA effects we observed in total. PPARγ agonists have been demonstrated to stimulate lipid synthesis in a mammary epithelial cell line [14]. We observed lower PPARγ2 mRNA abundance at 42 and at 105 DIM in the animals treated with CLA than in the cows receiving the control fat supplement. The mammary gland is an important target for CLA; as discussed by Bauman et al. [24], milk fat depression by CLA treatment is well established and was observed in the animals from the two trials herein, too [26], [29]. PPARγ was suggested by Kadegowda et al. [14] to be an important regulator of milk fat synthesis. It was shown that in parallel to PPARγ, lipogenic genes like stearoyl-CoA desaturase, diacylglycerol *O*-acyltransferase 2 and fatty acid synthase were down regulated during the peripartal period [89]. We speculate that reduced receptor capacity of the PPARγ2 isoform might be responsible for milk fat depression by CLA.

### CLA supplementation and systemic insulin sensitivity

For estimating the systemic IS, hyperinsulinaemic euglycaemic glucose clamps (HEC) are considered as “gold standard”, however, HEC can not realistically be accomplished at all days sampling was targeted. We used a surrogate marker, i.e. RQUICKI that can be accessed from individual more frequently obtained blood samples. The validity of the RQUICKI index was confirmed in dairy cows using data from previous clamp studies [90]. No CLA effects were observed on NEFA and BHBA concentrations [28], nevertheless, the RQUICKI index was reduced by 8% during the supplementation period and by 17% during the depletion period after 182 DIM in CLA treated cows vs. control cows indicating a reduction of IS by CLA. This was mainly attributable to greater insulin concentrations in CLA treated animals. Comparable effects on insulin concentrations using a similar CLA mixture were also observed in a mouse model, in which the results were explained by an increase in ß-cell mass and number [91]. In contrast to our results, Hötger et al. [25] observed no increase of insulin during supplementation with CLA for 9 weeks in dairy cows during their second lactation. The role of CLA as a ligand for the free fatty acid receptor 1 increasing glucose stimulated insulin secretion [22] might at least partly account for the insulin-increasing effects of CLA we observed in pluriparous cows. In addition, signalling by this receptor could also increase incretins, leading to insulin secretion [92]. Differences in the expression of free fatty acid receptor 1 on pancreatic ß-cells and on enteroendocrine cells between primiparous vs. pluriparous cows could not be excluded. Parity seems important in this context since we did not see the CLA effect on insulin or RQUICKI when studying an additional set of 10 primiparous cows in Trial 1 (5 cows each supplemented with either CLA or control fat) from which only blood samples were assessed (data not shown).

## Conclusion

Studying primiparous and pluriparous cows, we observed changes in the mRNA abundance of different modulators of IS like the ADIPOQ and LEP system in different tissues focusing on AT and liver. Besides TNF-α, the abundance of all mRNAs of interest decreased after parturition in s.c. AT of pluriparous cows but not in liver where most of the mRNAs increased except IL-6. By our study we have shown in primiparous cows that individual AT depots of cows may differentially influence the regulation of IS. Based on the number of changes in different tissues, the impact of different genes on metabolism during lactation may also vary. Most changes were observed in case of ADIPOQR1 and TNF-α. Most of the differences occurred in retroperitoneal AT. Differences between different AT depots, even of s.c. origin should be considered for further studies. Finally, we showed that PPARγ2, as a regulator of milk fat synthesis in cattle, is reduced in mammary gland by CLA supplementation which might be a direct link to CLA induced milk fat depression.
